# Hepatitis B susceptibility and subsequent vaccination in priority populations across an Australian sentinel surveillance network, 2017*–*2023

**DOI:** 10.1017/S0950268825100241

**Published:** 2025-07-30

**Authors:** Leila Bell, Virginia Pilcher, Elly Layton, Victoria Polkinghorne, Jason Asselin, Anna Wilkinson, Joseph Doyle, Phillip Read, Mish Pony, Stella Pendle, Wayne Dimech, Mark Stoové, Basil Donovan, Jessica Howell, Margaret E Hellard

**Affiliations:** 1 Disease Elimination, Burnet Institute, Melbourne, VIC, Australia; 2 School of Public Health and Preventive Medicine, Monash University, Melbourne, VIC, Australia; 3 Melbourne School of Population and Global Health, University of Melbourne, Melbourne, VIC, Australia; 4 Department of Infectious Diseases, The Alfred and Monash University, Melbourne, VIC, Australia; 5 Sexual Health and Blood Borne Virus Services, South Eastern Sydney Local Health District, NSW Health, NSW, Australia; 6 Scarlet Alliance, Australian Sex Workers Association, Newtown, NSW, Australia; 7 Microbiology Department, Australian Clinical Labs, Bella Vista, NSW, Australia; 8 Scientific and Business Relations, National Reference Laboratory, Melbourne, VIC, Australia; 9 Kirby Institute, University of New South Wales, Sydney, NSW, Australia; 10 Department of Gastroenterology, St Vincent’s Hospital and University of Melbourne, Melbourne, VIC, Australia; 11 Doherty Institute and School of Population and Global Health, University of Melbourne, Melbourne, VIC, Australia

**Keywords:** hepatitis B vaccination, hepatitis B virus, hepatitis B susceptibility, vaccination coverage, priority populations, sentinel surveillance system

## Abstract

Hepatitis B virus vaccination is currently recommended in Australia for adults at an increased risk of acquiring infection or at high risk of complications from infection. This retrospective cohort study used data from an Australian sentinel surveillance system to assess the proportion of individuals who had a recorded test that indicated being susceptible to hepatitis B infection in six priority populations, as well as the proportion who were then subsequently vaccinated within six months of being identified as susceptible. Priority populations included in this analysis were people born overseas in a hepatitis B endemic country, people living with HIV, people with a recent hepatitis C infection, gay, bisexual and other men who have sex with men, people who have ever injected drugs, and sex workers. Results of the study found that in the overall cohort of 43,335 individuals, 14,140 (33%) were identified as susceptible to hepatitis B, and 5,255 (37%) were subsequently vaccinated. Between 26% and 33% of individuals from priority populations were identified as susceptible to hepatitis B infection, and the proportion of these subsequently vaccinated within six months was between 28% and 42% across the groups. These findings suggest further efforts are needed to increase the identification and subsequent vaccination of susceptible individuals among priority populations recommended for hepatitis B vaccination, including among people who are already engaged in hepatitis B care.

## Introduction

Hepatitis B is a vaccine-preventable blood-borne viral infection that can cause liver damage, leading to liver failure, liver cirrhosis, and hepatocellular carcinoma [2], therefore generating a significant burden of disease in Australia and globally. The risk of developing chronic infection is much higher (approximately 90%) when exposure happens before one year of age, compared with a 30% risk at under five and around 5% thereafter [3]. Therefore, the focus for prevention has primarily been on childhood infection, with hepatitis B vaccines first registered for use in Australia in the early 1980s, and a universal infant vaccination program in place since 2000 [4]. Infant hepatitis B vaccination is funded by the Commonwealth government under the National Immunisation Program and consists of a birth dose plus three additional doses at two, four, and six months of age [1]. Rates of infant vaccination for hepatitis B in Australia are high, with vaccine coverage for children over 24 months of age consistently exceeding the national target of 95% from 2017 until 2021 [5]. Although birth dose is not generally reported at the national level, in New South Wales, over 92% of babies were given the hepatitis B vaccine at birth annually from 2007 to 2022 [6]. In 2017, in Victoria, 85% of infants received the hepatitis B birth dose within 24 h and, from 2009 to 2017, 89% of infants received the hepatitis B birth dose within seven days [7].

A total of 200,385 people in Australia were estimated to be living with chronic hepatitis B in 2021, with an estimated 140,317 people, or 76% of people, living with chronic hepatitis B infection born overseas [5]. National prevalence estimates are 0.8%, with higher prevalence among people born in Northeast Asia (5%), Southeast Asia (4%), and Sub-Saharan Africa (2%), as well as people who inject drugs (3%), and gay and bisexual men (2%) [5]. Among new hepatitis B notifications, the highest rates in 2021 were seen among people aged 35–39, with declining rates over the past decade among younger age groups and consistently higher rates among males than females [5]. The Australian Technical Advisory Group on Immunisation (ATAGI) current guidelines recommend hepatitis B vaccination of individuals who belong to a priority population at an increased risk of infection and who are not immune to hepatitis B. These guidelines are supported by the Australian consensus recommendations for the management of hepatitis B [1, 8, 9]. Since hepatitis B can be transmitted through injecting drug use and sexual contact, as well as household contacts, priority populations include people born in a hepatitis B endemic country; people living with human immunodeficiency virus (HIV); people with a history of hepatitis C infection; gay, bisexual and other men who have sex with men (GBMSM); people who inject drugs; and sex workers [1, 8]. Adult vaccination is a three-dose schedule within six months for full protection [1].

There is no specific adult vaccination coverage target in the latest draft Australian National Hepatitis B Strategy [4]. However, engagement with and vaccination of priority populations, especially people born in hepatitis B endemic countries, is specified as a key area for action in the strategy [4]. In contrast to infant vaccination, adult vaccination is funded by jurisdictional government, with variations in funding and priority groups across jurisdictions, making it difficult to monitor vaccine uptake [10, 11]. While it is known that surface antibody titres may wane over time without loss of immunity [9], it is reasonable to vaccinate people from priority populations if they are identified as hepatitis B surface antibody (anti-HBs) negative, particularly if they were born after introduction of universal vaccination in their country of birth.

This study aims to describe the hepatitis B susceptibility and subsequent vaccination coverage for populations identified as priorities for hepatitis B testing and vaccination, disaggregated by age, sex, and hepatitis B endemicity in their country of birth, in a sentinel surveillance system in Australia. The study quantifies the number of individuals who were identified as hepatitis B susceptible and were subsequently vaccinated within six months.

## Methods

### Study design

This retrospective cohort study analysed electronic medical data. Data were routinely extracted from patient management systems by the Australian Collaboration for Coordinated Enhanced Sentinel Surveillance of Sexually Transmissible Infections and Blood Borne Viruses (ACCESS). ACCESS is a sentinel surveillance system of over 100 health services (sites) around Australia established to monitor blood-borne viruses (BBVs) including HIV; hepatitis B virus; hepatitis C virus (HCV); and sexually transmissible infections (STIs) including chlamydia, gonorrhoea, and syphilis. ACCESS collates clinical and demographic data from participating sexual health clinics, general practices, hospital clinics, community health clinics, drug and alcohol services, and pathology services. Data are non-identifiable but contain anonymous unique identifiers, allowing patient data to be linked over time, both within and between participating sites. Detailed methods for ACCESS have been published elsewhere [12].

Participating ACCESS sites specialise in the care of BBV and STI priority populations. For this study, we included a subset of clinics with data available on hepatitis B testing and vaccination across the study period (1 January 2017 to 30 June 2023 for testing, and vaccination data up to 31 December 2023). Individuals were included if they attended one of the included clinics, were aged 16 years or over at the time of testing, and if there was sufficient data to identify them as being part of a priority population. Having sex recorded as male or female was a study eligibility criterion because patient sex is the most complete data field entered into electronic medical records. Additionally, to be included in the analysis, individuals needed to have evidence of testing for (1) hepatitis B surface antigen (HBsAg), (2) hepatitis B core antibody (anti-HBc), and (3) anti-HBs within a 30-day period from the first testing episode (i.e. if HBsAg negative on 1 January 2017, the individual would be included if they had an anti-HBc and anti-HBs test by 30 January 2017) at an included ACCESS site during the study period. An individual’s first identified test for hepatitis B during the study period was used for the analysis.

The priority populations included in the analysis were (1) **people born overseas in an hepatitis B endemic country**, defined as being born in a country other than Australia where the 2022 hepatitis B prevalence estimate is >2% according to the Centre for Disease Analysis Foundation’s Polaris Observatory [13] or, if not available from Polaris, from the Global Health Obesrvatory [14]; (2) **people living with HIV**, defined as having evidence of a HIV diagnosis or monitoring test result recorded within ACCESS; (3) **people with a recent hepatitis C infection**, defined as having a positive hepatitis C ribonucleic acid (RNA) test result recorded within ACCESS in the 12 months prior to hepatitis B testing; (4) **GBMSM**, defined as (i) ever being recorded as male and gay or bisexual in ACCESS or (ii) being male and ever having a rectal swab test result for chlamydia or gonorrhoea recorded in ACCESS [15]; (5) **people who have ever injected drugs**, defined as (i) a self-reported history of injecting drug use on behavioural survey conducted only at sexual health clinics or (ii) having any record in ACCESS of being prescribed opioid agonist therapy; (6) current or past **sex workers**, defined as a self-reported history of current or past sex work on routine behavioural surveys conducted at sexual health clinics. Other populations recommended for vaccination by ATAGI were not included in this analysis because they cannot be reliably identified in the dataset.

### Analysis

For each priority population cohort, we calculated the number of individuals who were tested for hepatitis B, the number and proportion of individuals tested identified as susceptible to hepatitis B, and the number and proportion of individuals who had a hepatitis B vaccine within the six months after being identified as susceptible. A six-month cut-off was used as a reasonable clinical expectation for vaccination to happen in response to the test result of being hepatitis B susceptible. Hepatitis B susceptibility was defined as all HBsAg, anti-HBc, and anti-HBs assays (including having an anti-HBs titre <10 mIU/mL) being negative, acknowledging that a proportion of participants may have been vaccinated for hepatitis B in the past and experienced decreasing surface antibody titres over time. However, the fact that testing was performed suggests the clinician and the participant were unaware or uncertain of this occurring.

Hepatitis B vaccination was defined as (i) the prescription or evidence of administration of at least one dose of hepatitis B vaccine, occurring on or after the date the person was assigned to be susceptible to hepatitis B on testing or (ii) a positive anti-HBs assay (titre ≥10 mIU/mL) and negative HBsAg and anti-HBc assays, occurring any time within six months after the date the person was identified as susceptible to hepatitis B on testing indicating anti-HBs seroconversion post vaccination (noting a small proportion may not have mounted an antibody response to the vaccine and be non-responders). Due to ACCESS being able to link individuals across participating services, the site where the hepatitis B vaccination or subsequent serology occurred did not need to be the site where initial testing occurred, as long as the site participated in ACCESS [12]. Each cohort was then stratified by age, sex, and place of birth.

Ethics approval for ACCESS was granted by the human research ethics committees of the Alfred Hospital (248/17), the University of Tasmania (H0010220), and the Menzies School of Health Research (08/047). This study is reported in accordance with the strengthening the reporting of observational studies in epidemiology (STROBE) statement [16].

## Results

**Figure 1.**
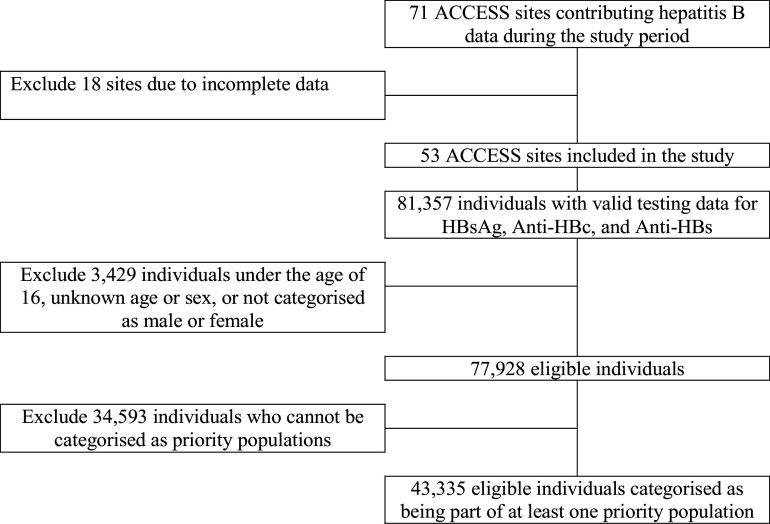
Flow diagram of selection steps.

Among the 53 clinics included in the analyses from 1 January 2017 to 30 June 2023, there were 81,357 individuals with valid testing results for HBsAg, Anti-HBc, and Anti-HBs conducted. A total of 3,429 individuals were excluded because of missing age or sex data, being under 16 years of age, and a further 34,593 individuals excluded because they did not have data that indicated they were part of a priority population, leaving a total of 43,335 eligible individuals that meet the study selection criteria (refer to Figure 1 for a flow diagram of selection steps). Among individuals included, 72% belonged to one priority population only, 24% belonged to two priority populations, and the remaining 4% belonged to three or more priority populations. The largest priority population represented in this study was gay and bisexual men (32,236; 74%), followed by people born in a hepatitis B endemic country (7,751: 18%), people who have ever injected drugs (6,300; 15%), and people living with HIV (5,622; 13%). The analysis also included people who self-reported ever engaging in sex work (3,860; 9%) and people with recent hepatitis C infection (1,581; 4%). Among total individuals, 37,313 (86%) were male and patient age ranged from 16 to 93 years, with a median age of 35 years (interquartile range: 28–47).

Among the 43,335 individuals included in the analysis, a total of 14,140 (33%) were identified as susceptible to hepatitis B infection on serology. The proportion of hepatitis-B-susceptible people was lower among people born in a hepatitis B endemic country (28%, 2,184/7,751) compared to those born in Australia (34%, 7,250/21,033), those born in a non-endemic country (33%, 2,031/6,231), or those with either no country of birth recorded or in a country with unknown hepatitis B prevalence (32%, 2,675/8,320). Among people tested, 36% (4,884/13,533) of individuals in the 16–29 years age group were identified to be susceptible compared with all other age groups above 30 years, which ranged between 31% and 32%. Males and females had similar susceptibility to hepatitis B in this study. A description of the full cohort of individuals included in this analysis can be seen in Table 1.

**Table 1.** The numbers and proportions of people identified as being part of a priority population and tested for hepatitis B in the ACCESS surveillance system, 2017 to 2023; *N* = 43,335 people tested

Age in years	
16–29	13,533 (31%)
30–39	12,915 (30%)
40–49	8,261 (19%)
50–59	5,641 (13%)
60+	2,985 (7%)
Sex	
Male	37,313 (86%)
Female	6,022 (14%)
Place of birth	
Born in Australia	21,033 (49%)
Born overseas – HBV endemic country	7,751 (18%)
Born overseas – non-HBV endemic country	6,231 (14%)
Missing or country with unknown HBV prevalence	8,320 (19%)
Proportion testing positive	
HBsAg +	842 (2%)
Anti-HBc +	7,219 (17%)
Anti-HBs +	27,445 (63%)
Proportion in each priority group	
People with human immunodeficiency virus (HIV) infection	5,622 (13%)
People with hepatitis C virus (HCV) infection	1,581 (4%)
Gay and bisexual men	32,236 (74%)
People who inject drugs	6,300 (15%)
Sex workers	3,860 (9%)
Number of individuals in multiple priority groups	
1	31,277 (72%)
2	10,342 (24%)
3	1,487 (3%)
4–6	229 (1%)
	*N* (percent of full cohort, *N* = 43,335)
Age in years	
16–29	13,533 (31%)
30–39	12,915 (30%)
40–49	8,261 (19%)
50–59	5,641 (13%)
60+	2,985 (7%)
Sex	
Male	37,313 (86%)
Female	6,022 (14%)
Place of birth^a^	
Born in Australia	21,033 (49%)
Born overseas – HBV endemic country	7,751 (18%)
Born overseas – non-HBV endemic country	6,231 (14%)
Missing or country with unknown HBV prevalence	8,320 (19%)
Proportion testing positive	
HBsAg +	842 (2%)
Anti-HBc +	7,219 (17%)
Anti-HBs +	27,445 (63%)
Proportion in each priority group	
People with human immunodeficiency virus (HIV) infection	5,622 (13%)
People with hepatitis C virus (HCV) infection	1,581 (4%)
Gay and bisexual men	32,236 (74%)
People who inject drugs	6,300 (15%)
Sex workers	3,860 (9%)
Number of individuals in multiple priority groups	
1	31,277 (72%)
2	10,342 (24%)
3	1,487 (3%)
4–6	229 (1%)

a Hepatitis B endemic countries: Albania, Angola, Bangladesh, Belarus, Bosnia and Herzegovina, Botswana, Bulgaria, Cambodia, Cameroon, Central African Republic, China (Mainland), Comoros, Cook islands, Cote d’Ivoire, Democratic People’s Republic of Korea, Eritrea, Ethiopia, Gabon, Ghana, Guinea, Guyana, Hong Kong (SAR of China), Indonesia, Iraq, Jamaica, Kenya, Kiribati, Kyrgyzstan, Lao People’s Democratic Republic, Liberia, Malawi, Maldives, Mali, Mauritania, Mauritius, Moldova, Mongolia, Mozambique, Myanmar, Namibia, Nigeria, Oman, Papua New Guinea, Philippines, Republic of Congo, Republic of Korea, Samoa, Senegal, Seychelles, Sierra Leone, Solomon Islands, Somalia, South Africa, Sudan, Swaziland, Syria, Taiwan, Tajikistan, Thailand, Timor-Leste, Togo, Tonga, Tunisia, Turkmenistan, Uruguay, Vanuatu, Vietnam, Zimbabwe. Hepatitis B non-endemic countries: Afghanistan, Algeria. Argentina, Armenia, Australia, Austria, Azerbaijan, Bahamas, Bahrain, Barbados, Belgium, Belize, Bhutan, Brazil, Brunei Darussalam, Burundi, Canada, Chile, Colombia, Costa Rica, Croatia, Cuba, Cyprus, Czechia, Denmark, Dominican Republic, Ecuador, Egypt, El Salvador, Estonia, Fiji, Finland, France, The Gambia, Georgia, Germany, Greece, Guatemala, Haiti, Hungary, India, Iran, Ireland, Israel Italy, Japan, Jordan, Kazakhstan, Kuwait, Latvia, Lebanon, Libya, Lithuania, Malaysia, Malta, Mexico, Morocco, Nepal, Netherlands, New Zealand, North Macedonia, Norway, Pakistan, Panama, Paraguay, Peru, Poland, Portugal, Rwanda, Saudi Arabia, Serbia, Singapore, Slovakia, Slovenia, Spain, Sri Lanka, St. Lucia, Sweden, Switzerland, Trinidad and Tobago, Türkiye, Uganda, Ukraine, United Arab Emirates, United Kingdom, United States of America, Uzbekistan, Zambia. Unknown endemicity: Bermuda, Guam, Macau (SAR of China), New Caledonia, Puerto Rico, Yugoslavia.

Among individuals identified as susceptible, 5,255 (37%) had evidence of vaccination within six months of their initial test (Table 1). Vaccination of people identified as susceptible was higher among people born in endemic countries (42%, 916/2,184) and people born in non-endemic countries (42%, 854/2,031) compared to people born in Australia (36%, 2,586/7,250). Males had a higher rate of vaccination, with 38% (4,637/12,169) of men identified as susceptible subsequently vaccinated within six months, compared to 31% (618/1,971) of women. Younger age groups were more likely to have evidence of vaccination than older groups, with 39% (1,895/4,884) and 40% (1,582/3,963) people aged 16–29 years and 30–39 years having evidence of vaccination, respectively, with a decreasing trend for older age groups.

**Figure 2.**
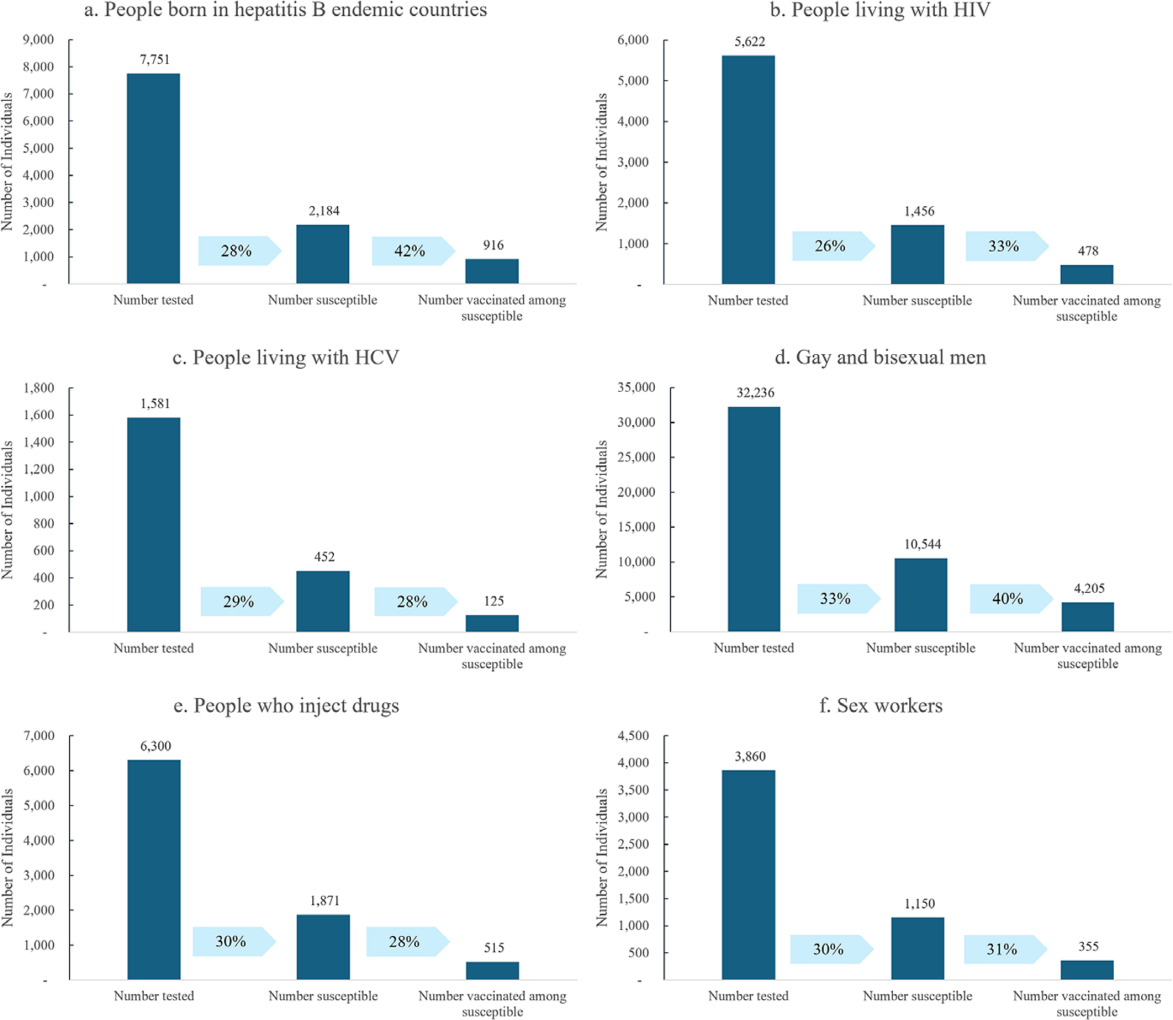
Cascade of individuals receiving hepatitis B testing, individuals identified as susceptible to hepatitis B, and susceptible individuals who received subsequent hepatitis B vaccination in six months following testing, by priority population.

Of the 5,622 (13%) people living with HIV, 1,456 (26%) were identified as susceptible, and 478 (33%) of those susceptible were vaccinated. Of the 1,581 (4%) people with a recent history of hepatitis C infection, 452 (29%) were identified as susceptible and 125 (28%) of those susceptible were vaccinated with the hepatitis B vaccine. Of the 32,236 (74%) GBMSM, 10,544 (33%) were identified as susceptible, and 4,205 (40%) of those susceptible were vaccinated. Of the 6,300 (15%) people who had ever injected drugs, 1,871 (30%) were identified as susceptible, and 515 (28%) of those susceptible were vaccinated. Of the 3,860 (9%) current or past sex workers, 1,150 (30%) were identified as susceptible, and 355 (31%) of those susceptible were vaccinated. The susceptibility and vaccination of these priority populations are displayed in Figure 2 and presented stratified by age, gender, and place of birth in Table 2.

**Table 2.** The numbers and proportions of people identified as susceptible to hepatitis B who received subsequent hepatitis B vaccination in the six months following testing by age, sex, and priority group in the ACCESS surveillance system, 2017 to 2023, *N* = 43,335 people tested

	Number of people tested for hepatitis B^a^ *n*	People tested for hepatitis B identified as susceptible to hepatitis B^b^ *n* (total susceptible/total tested%)	People susceptible to hepatitis B who were subsequently vaccinated within six months^c^ *n* (total vaccinated/total susceptible%)
Full cohort			
Total	43,335	14,140 (33%)	5,255 (37%)
Age groups (years)			
16–29	13,533	4,884 (36%)	1,895 (39%)
30–39	12,915	3,963 (31%)	1,582 (40%)
40–49	8,261	2,613 (32%)	894 (34%)
50–59	5,641	1,768 (31%)	593 (34%)
60+	2,985	912 (31%)	291 (32%)
Sex			
Male	37,313	12,169 (33%)	4,637 (38%)
Female	6,022	1,971 (33%)	618 (31%)
Place of birth **^d^**			
Born in Australia	21,033	7,250 (34%)	2,586 (36%)
Born overseas – hepatitis B endemic country	7,751	2,184 (28%)	916 (42%)
Born overseas – non-hepatitis B endemic country	6,231	2,031 (33%)	854 (42%)
Missing or country of unknown endemicity	8,320	2,675 (32%)	899 (34%)
People born in hepatitis B virus endemic countries			
Total	7,751	2,184 (28%)	916 (42%)
Age in years			
16–29	3,057	1,086 (36%)	465 (43%)
30–39	2,673	663 (25%)	297 (45%)
40–49	1,254	258 (21%)	98 (38%)
50–59	534	121 (23%)	38 (31%)
60+	233	56 (24%)	18 (32%)
Sex			
Male	4,999	1,407 (28%)	633 (45%)
Female	2,752	777 (28%)	283 (36%)
People with human immunodeficiency virus (HIV) infection			
Total	5,622	1,456 (26%)	478 (33%)
Age in years			
16–29	556	176 (32%)	81 (46%)
30–39	1,246	327 (26%)	143 (44%)
40–49	1,439	393 (27%)	117 (30%)
50–59	1,517	374 (25%)	97 (26%)
60+	864	186 (22%)	40 (22%)
Sex			
Male	5,166	1,303 (25%)	425 (33%)
Female	456	153 (34%)	53 (35%)
Place of birth^d^			
Born in Australia	3,171	893 (28%)	279 (31%)
Born overseas – hepatitis B endemic country	725	156 (22%)	59 (38%)
Born overseas – non-hepatitis B endemic country	891	206 (23%)	79 (38%)
Missing or country of unknown endemicity	835	201 (24%)	61 (30%)
People with hepatitis C virus infection			
Total	1,581	452 (29%)	125 (28%)
Age in years			
16–29	93	20 (22%)	8 (40%)
30–39	451	124 (27%)	39 (31%)
40–49	570	184 (32%)	50 (27%)
50–59	360	102 (28%)	25 (25%)
60+	107	22 (21%)	3 (14%)
Sex			
Male	1,118	133 (12%)	78 (59%)
Female	463	141 (30%)	47 (33%)
Place of birth^d^			
Born in Australia	511	165 (32%)	53 (32%)
Born overseas – hepatitis B endemic country	33	6 (18%)	2 (33%)
Born overseas – non-hepatitis B endemic country	61	17 (28%)	6 (35%)
Missing or country of unknown endemicity	976	264 (27%)	64 (24%)
Gay and bisexual men			
Total	32,236	10,544 (33%)	4,205 (40%)
Age in years			
16–29	10,591	3,739 (35%)	1,534 (41%)
30–39	9,666	2,969 (31%)	1,254 (42%)
40–49	5,528	1,777 (32%)	673 (38%)
50–59	4,114	1,331 (32%)	488 (37%)
60+	2,337	728 (31%)	256 (35%)
Place of birth^d^			
Born in Australia	17,642	6,002 (34%)	2,292 (38%)
Born overseas – hepatitis B endemic country	3,548	927 (26%)	458 (49%)
Born overseas – non-hepatitis B endemic country	5,565	1,750 (31%)	769 (44%)
Missing or country of unknown endemicity	5,481	1,865 (34%)	686 (37%)
People who inject drugs			
Total	6,300	1,871 (30%)	515 (28%)
Age in years			
16–29	884	263 (30%)	77 (29%)
30–39	2,013	630 (31%)	188 (30%)
40–49	2,068	655 (32%)	170 (26%)
50–59	1,050	265 (25%)	69 (26%)
60+	285	58 (20%)	11 (19%)
Sex			
Male	4,715	1,344 (29%)	376 (28%)
Female	1,585	527 (33%)	139 (26%)
Place of birth^d^			
Born in Australia	3,253	1,058 (33%)	285 (27%)
Born overseas – hepatitis B endemic country	217	44 (20%)	20 (45%)
Born overseas – non-hepatitis B endemic country	446	103 (23%)	29 (28%)
Missing or country of unknown endemicity	2,384	666 (28%)	181 (27%)
Sex workers			
Total	3,860	1,150 (30%)	355 (31%)
Age in years			
16–29	1,530	519 (34%)	156 (30%)
30–39	1,298	354 (27%)	117 (33%)
40–49	678	179 (26%)	50 (28%)
50–59	282	70 (25%)	22 (31%)
60+	72	28 (39%)	10 (36%)
Sex			
Male	1,535	459 (30%)	144 (31%)
Female	2,325	691 (30%)	211 (31%)
Place of birth^d^			
Born in Australia	2,277	764 (34%)	216 (28%)
Born overseas – hepatitis B endemic country	853	102 (12%)	153 (52%)
Born overseas – non-hepatitis B endemic country	578	223 (39%)	70 (31%)
Missing or country of unknown endemicity	152	61 (40%)	16 (26%)

a defined as tested for HBsAg, Anti-HBc, and Anti-HBs.

b defined as negative for HBsAg, Anti-HBc, and Anti-HBs within a 30-day period from the first test.

c defined as evidence of at least one hepatitis B vaccine within six months of testing episode.

d Hepatitis B endemic countries: Albania, Angola, Bangladesh, Belarus, Bosnia and Herzegovina, Botswana, Bulgaria, Cambodia, Cameroon, Central African Republic, China (Mainland), Comoros, Cook islands, Cote d’Ivoire, Democratic People’s Republic of Korea, Eritrea, Ethiopia, Gabon, Ghana, Guinea, Guyana, Hong Kong (SAR of China), Indonesia, Iraq, Jamaica, Kenya, Kiribati, Kyrgyzstan, Lao People’s Democratic Republic, Liberia, Malawi, Maldives, Mali, Mauritania, Mauritius, Moldova, Mongolia, Mozambique, Myanmar, Namibia, Nigeria, Oman, Papua New Guinea, Philippines, Republic of Congo, Republic of Korea, Samoa, Senegal, Seychelles, Sierra Leone, Solomon Islands, Somalia, South Africa, Sudan, Swaziland, Syria, Taiwan, Tajikistan, Thailand, Timor-Leste, Togo, Tonga, Tunisia, Turkmenistan, Uruguay, Vanuatu, Vietnam, Zimbabwe. Hepatitis B non-endemic countries: Afghanistan, Algeria. Argentina, Armenia, Australia, Austria, Azerbaijan, Bahamas, Bahrain, Barbados, Belgium, Belize, Bhutan, Brazil, Brunei Darussalam, Burundi, Canada, Chile, Colombia, Costa Rica, Croatia, Cuba, Cyprus, Czechia, Denmark, Dominican Republic, Ecuador, Egypt, El Salvador, Estonia, Fiji, Finland, France, The Gambia, Georgia, Germany, Greece, Guatemala, Haiti, Hungary, India, Iran, Ireland, Israel Italy, Japan, Jordan, Kazakhstan, Kuwait, Latvia, Lebanon, Libya, Lithuania, Malaysia, Malta, Mexico, Morocco, Nepal, Netherlands, New Zealand, North Macedonia, Norway, Pakistan, Panama, Paraguay, Peru, Poland, Portugal, Rwanda, Saudi Arabia, Serbia, Singapore, Slovakia, Slovenia, Spain, Sri Lanka, St. Lucia, Sweden, Switzerland, Trinidad and Tobago, Türkiye, Uganda, Ukraine, United Arab Emirates, United Kingdom, United States of America, Uzbekistan, Zambia. Unknown endemicity: Bermuda, Guam, Macau (SAR of China), New Caledonia, Puerto Rico, Yugoslavia.

## Discussion

In a cohort of 43,335 people in at least one priority group where hepatitis B vaccination is recommended, approximately a third of the cohort were identified as being susceptible to hepatitis B, and of those, fewer than 40% had evidence of subsequent vaccination within six months. Within this priority population cohort, a lower proportion of people born in hepatitis B endemic countries were identified as susceptible to hepatitis B (28%) compared to people in priority populations born in Australia or in non-endemic countries (34% and 33%, respectively). Vaccination after detection of susceptibility was highest among people born in hepatitis B endemic countries and gay and bisexual men, with 42 and 40% being vaccinated within six months, respectively. Our results suggest that despite guidelines recommending vaccination for all priority groups as early as 2013 (and since 1986 for gay and bisexual men), a significant proportion of people identified as belonging to priority populations and tested were susceptible to hepatitis B and many did not undergo timely vaccination, despite their engagement in hepatitis B care through initial testing and the widespread availability of hepatitis B vaccine in Australia.

These results are similar to a study in London where 40% of individuals with at least one risk factor indicating adult vaccination and approximately 25% of individuals with two or more risk factors were identified as unvaccinated [17]. In the United States of America, it was found that 55% of adults at higher risk of hepatitis B infection were susceptible to hepatitis B, higher than what was found in our study [18]. Challenges to adult vaccination uptake can be classified into four factors – personal beliefs around vaccination, financial, logistics, and factors related to healthcare workers such as clinician awareness or recommendations [19]. While it was beyond the scope of this study to examine the reason for hepatitis B vaccine delivery rates, it may be due to differences in provider awareness of vaccination recommendations or emphasis on the importance of vaccination for different priority populations. There are also known differences in engagement in care, for example, GBMSM may be more routinely engaged in care due to engagement in sexual health screening, while people who inject drugs may experience more interruptions in care [20, 21]. In Victoria, for example, there was a targeted effort in 2017–2018 to increase hepatitis B vaccination particularly among GBMSM and people who inject drugs as a broader effort to increase vaccination for hepatitis B, meningococcal disease and human papillomavirus, during a hepatitis A outbreak [22]. Previous studies have found that financial incentives and accelerated vaccine schedules may increase the likelihood of completing a full course of hepatitis B vaccination among people who inject drugs [23, 24], while there is some evidence that financial incentives may incentivise adult vaccination more broadly [25]. Further research into the barriers and enablers of hepatitis B vaccine delivery and uptake among susceptible adults in priority populations is crucial to develop strategies to increase vaccination. Additionally, because policies around adult vaccination and financing are determined by states and territories, there is a need for improved visibility of adult hepatitis B vaccination data across jurisdictions to monitor national progress.

Models are available to estimate progress towards global and national targets [26] and have found that Australia is not on track to meet the global target of a 90% reduction in incident hepatitis B infections by 2030 [27]. Additional strategies are required to progress towards this target, including improved vaccination of adults identified as susceptible to hepatitis B infection. Additionally, in order to reach the global target of a reduction in hepatitis B related deaths by 65% by 2030 [27], it is critical to increase screening of adults in Australia and then ensure access to affordable vaccination for all susceptible persons as well as linkage to care for people identified as living with hepatitis B [28]. In Europe, 20 countries have policies recommending hepatitis B vaccination among adults, with some countries recommending universal vaccination [29]. In 2021, universal vaccination of susceptible adults aged 19–59 years was recommended by the Advisory Committee on Immunisation Practices in the United States of America after an increase in acute hepatitis B infection was reported in some age groups [30]. Expanded hepatitis B immunisation among adults was found to be cost-effective in several settings, including the USA [23], the Netherlands [31], and among young adults in China [32].

There are several limitations to this study that should be considered when interpreting the above results. First, it is known that anti-HBs titres reduce over time after vaccination, without necessarily conferring a loss of immunity [9]. If an individual was identified as susceptible to infection on serology in our study, but had a documented history of appropriate vaccination, some providers may have chosen not to provide further vaccination. Hence, we may have overestimated ongoing susceptibility to hepatitis B. Conversely, including evidence of one vaccination to imply immunity, as was done in this study, may overestimate immunity [8], as the participant may not have completed a full course of vaccination. Additionally, it is known that people living with HIV may have suboptimal immunological response to hepatitis B vaccination, underestimating vaccine delivery [33]. Second, many individuals who receive care at ACCESS sites and are members of a priority population for hepatitis B screening would not have been tested for hepatitis B serology because they had evidence of prior testing or vaccination. As per clinical guidelines, clinicians test clients who might not be immune (e.g. no evidence of vaccination or previous infection), so it is not possible to assess proportion testing among priority populations in the full ACCESS cohort. Third, this study was only able to capture vaccinations that were prescribed or administered or follow-up serology that was performed, at a site that participates in ACCESS. It is possible that after being identified as susceptible to hepatitis B at an ACCESS site, individuals received their vaccination or had serology performed elsewhere, leading to an underestimate of vaccine uptake. Fourth, importantly, this study analysed data from a sentinel surveillance network and therefore results cannot necessarily be extrapolated to all of Australia. ACCESS sites are deliberately selected to have over representation of people at risk of BBVs and STIs, including GBMSM, people who inject drugs, and sex workers. Fifth, this study may have underestimated the level of hepatitis B susceptibility in these populations if a person had no evidence of hepatitis B testing at an ACCESS service; while they may have been tested elsewhere, they may never have been tested for hepatitis B and thus be at risk of infection. Sixth, individuals seeking care at ACCESS sites who did not have a recorded sex as male or female were excluded because of the inconsistent recording of non-binary sex and/or diverse gender identities within electronic medical records. Additionally, transgender and gender diverse people may have their sex recorded as either male or female on medical records, but not necessarily in a consistent way [34]. Therefore, these results are not generalisable to transgender or gender diverse people, who may also have different hepatitis B risks [35]. Finally, using the estimated population prevalence of 2% to classify endemicity of a country does not reflect recent decreases in prevalence among people born after the introduction of childhood immunisation programmes in a specific country. While this is true, the consensus guidelines recommend screening among people born in countries with 2% endemicity, and therefore, this was used as a cut-off [9]. Notwithstanding these limitations, the data presented here provide important insights into vaccination uptake among priority populations at greatest risk of hepatitis B infection in Australia.

## Conclusion

Despite clear national guidelines recommending hepatitis B vaccination in priority populations in Australia and national strategies aiming for hepatitis B elimination, results from our study suggest a high proportion of tested individuals from priority populations are susceptible to hepatitis B infection. Having been engaged in hepatitis B care and identified as being susceptible, a high proportion then had no evidence of timely vaccination to reduce the risk of hepatitis B infection. The hepatitis B vaccine is safe and effective, is widely available throughout the country and is subsidised within the Australian health system and available for free for some priority populations in some jurisdictions. Work is needed to understand the barriers to people being tested for hepatitis B, as well as why people who are identified as being at risk of infection do not get vaccinated. If Australia is to achieve its hepatitis B elimination targets, including reducing hepatitis B incidence, it is critical that high levels of vaccination coverage are achieved, particularly in people known to be at the highest risk of infection.

## Data Availability

Data used in this study are from the ACCESS of BBVs and STIs (ACCESS) and are not publicly available. The data can be made available upon reasonable request via ACCESS Data Management https://accessproject.org.au/contact.
